# Correlation of Chimerism with Acute Graft-versus-Host Disease in Rats following Liver Transplantation

**DOI:** 10.4061/2011/947150

**Published:** 2011-05-17

**Authors:** Fei Xue, Wei Chen, Xue-Li Bai, Guo-Dong Xu, Liang Liang, Ting-Bo Liang

**Affiliations:** ^1^Department of Hepatobiliary and Pancreatic Surgery, Key Laboratory of Combined Multi-Organ Transplantation of Ministry of Public Health, The First Affiliated Hospital, School of Medicine, Zhejiang University, 79 Qingchun Road, Hangzhou 310003, China; ^2^Department of Hepatobiliary and Pancreatic Surgery, Organ Transplantation Center, Henan Province People's Hospital, Zhengzhou 450003, Henan Province, China

## Abstract

The accurate diagnosis of acute graft-versus-host disease following liver transplantation (LTx-aGVHD) has been hampered. Chimerism appears in the majority of recipients after LT and its significance in the diagnosis of LTx-aGVHD has not been clearly established. To demonstrate the significance of chimerism on the diagnosis of LTx-aGVHD, we compared the change of chimerism in syngeneic LT recipients, semiallogeneic LT recipients, and LTx-aGVHD induced recipients. Chimerism in PBMCs following sex-mismatched LT was identified by real-time PCR based on a rat Y-chromosome-specific primer. All recipients in semiallogeneic group grew in a normal pattern. However, when 4 × 10^8^ donor splenocytes were transferred simultaneously during LT, the morbidity of lethal aGVHD was 100%. The chimerism appeared slightly higher in the semiallogeneic group than in the syngeneic LT group, but the difference was not significant. However, when the recipients developed lethal aGVHD after LT, chimerism in the PBMCs increased progressively, and even at an early time, a significant increase in chimerism was observed. In conclusion, high level chimerism correlated well with LTx-aGVHD, and detection of chimerism soon after transplantation may be of value in the diagnosis of LTx-aGVHD prior to the onset of symptoms.

## 1. Introduction

Acute graft-versus-host disease (aGVHD) is an uncommon but devastating complication that occurs in 1-2% of recipients after liver transplantation (LTx-aGVHD) in clinical course [1]. LTx-aGVHD symptoms usually appear 2 to 6 weeks after transplantation and are characterized by fever, skin rash, diarrhea, and pancytopenia. These symptoms may initially be difficult to differentiate from cytomegalovirus disease or drug-induced rash and pancytopenia. The accurate diagnosis of LTx-aGVHD has been hampered due to the lack of a sensitive and specific diagnostic test, and misdiagnosis may incur delayed treatment and diminish the chance of patient survival. Previous studies showed that the underdiagnosis of LTx-aGVHD is responsible for patients' mortality rates to be as high as 85% [2–4]. In our center, the liver transplantation program was established in 1993. We have identified 3 patients with LTx-aGVHD, and all died from infection, alimentary tract bleeding, or multiple organ failure [5]. 

Since LTx-aGVHD results from the engraftment of T lymphocytes associated with the liver graft, the demonstration of substantial donor T-lymphocyte chimerism may be of value in the diagnosis of this disease [6–17]. However, chimerism appears only transiently in the majority of patients in the early postoperative period after liver transplantation [18–22], and its significance in the diagnosis of LTx-aGVHD has not been clearly established. Furthermore, it is unknown whether macrochimerism commonly precedes symptomatic aGVHD. 

In this study, we investigate the level of chimerism in the peripheral blood that indicates the abnormal engraftment of donor lymphocytes in our previously established rat model with acute graft-versus-host disease following liver transplantation [23, 24] and found that this is an effective and senitive method in the early diagnosis of LTx-aGVHD.

## 2. Materials and Methods

### 2.1. Animals

Male Lewis (RT1^1^) rats weighing 200–300 g were used as donors. Female Lewis and (Lewis♀xBN♂)F1 (RT1^1/*n*^) rats of the same weight were used as recipients. Animals were purchased from Beijing Vital River Company. Both donors and recipients were housed in an animal facility under specific pathogen-free conditions and received humane care according to the National Institutes of Health guidelines. All surgical procedures were conducted under anesthesia using clean surgical instruments.

### 2.2. Liver Transplantation and Separation of Viable Splenocytes

Orthotopic liver transplantation was performed using the technique described by Kamada and Calne without the anastomosis of the hepatic artery [25]. The animals were allowed to recover in the operating suite, with free access to standard food and water. No antibiotic agents were used.

Viable splenocytes were separated using a slightly modified technique described by Kimura et al. [26]. Briefly, spleens from Lewis rats were minced and passed through 200-mesh stainless steel filters with 20 volumes of ice-cold RPMI-1640. Erythrocytes were removed by hypotonic lysis with sterile distilled water. The suspension was centrifuged at 300 × g for 10 minutes at 4°C. Splenocytes were resuspended in RPMI-1640, checked for viability by trypan blue dye exclusion, and counted.

### 2.3. LTx-aGVHD Induction and Animal Grouping

(LewisXBN)F1 recipients were injected with freshly prepared Lewis splenocytes via the femoral vein within 30 minutes from liver transplantation. Rats were divided as follows into four subgroups based on the numbers of splenocytes transferred. Group 1, syngeneic liver transplantation: Lewis rats received liver graft from the same strain without splenocyte transfusion. Group 2, semiallogeneic liver transplantation without splenocyte transfusion: liver transplantation was performed between Lewis rat (donor) and (LewisxBN)F1 rat (recipient) without splenocyte transfusion. Group 3, semiallogeneic liver transplantation with the transfusion of 2 × 10^8^ splenocytes: liver transplantation was performed between Lewis rat (donor) and (LewisxBN)F1 rat (recipient). Lewis splenocytes were adoptively transferred to the same recipient immediately after liver transplantation. Group 4, semiallogeneic liver transplantation with the transfusion of 4 × 10^8^ splenocytes: liver transplantation was performed between Lewis rat (donor) and (LewisxBN)F1 rat (recipient). Lewis splenocytes were adoptively transferred to the same recipient immediately after liver transplantation. Six rats in each group were used to monitor survival over 100 days. Peripheral blood was obtained every 4 days for 20 days after transplantation from six rats in groups 1, 2, and 4 and twelve rats in group 3. Recipient rats that developed aGVHD were sacrificed on day 16 for tissue sampling. Peripheral blood was obtained on day 50 from recipients that survived without developing aGVHD, and these rats were sacrificed on day 100 for tissue sampling.

### 2.4. Assessment of aGVHD

(1) Clinical course and animal survival. All animals were observed twice a day for typical aGVHD-related signs such as dermatitis, alopecia, weight loss, diarrhea, hunched posture, and cachexia [27]. The actuarial survival rate and mean time to death (mean survival time, MST) were calculated after observation for 100 days.

(2) Morphometric and histopathologic investigations. Tissue samples were taken at the time of death. Skin, small intestine, colon, and liver were pathologically evaluated. Each sample was fixed in 10% buffered neutral formalin, embedded in paraffin, and cut into 5 *μ*m thick sections, which were stained with hematoxylin-eosin (H&E). Slides were coded without reference to groups and examined in a blinded fashion by a pathologist. Abnormalities associated with aGVHD were observed [28, 29].

### 2.5. Real-Time PCR Analysis of Chimerism in Peripheral Blood Mononuclear Cells (PBMCs)

The level of chimerism in PBMCs after transplantation was determined using real-time PCR. Blood samples were collected in heparinized test tubes and were processed for analysis within 2 hours. PBMCs were isolated by density-gradient centrifugation over Ficoll-Hypaque (Shanghai Hengxin Chemical Reagent Co. Ltd., China). Genomic DNA was isolated from PBMCs by QIAamp DNA Mini Kit and QIAamp DNA Blood Mini Kit (Qiagen, Valencia, Calif, USA). All pairs of primers for real-time quantitative PCR were designed using the web-based program Primer 3 (http://frodo.wi.mit.edu/primer3/). Primers for SRY gene (forward primer 5′-CGAAGGGTTAAAGTGCCACAG-3′, reverse primer 5′-GTTCTTGGAGGACTGGTGTGC-3′, product of 150 bp) were designed to amplify all six SRY genes (SRY1, SRY2, SRY3, SRY3B, SRY3C, and SRY3bI) in order to increase the sensitivity of detection of male DNA. The total amount of rat genomic DNA or male genomic DNA was determined using the absolute quantification program of SDS2.0 software on an ABI7900 machine (Applied Biosystems, Foster City, Calif, USA). A series of dilutions of male rat genomic DNA (0.1 ng/*μ*L, 0.5 ng/*μ*L, 2.5 ng/*μ*L, 12.5 ng/*μ*L, and 50 ng/*μ*L) were used to construct standard curves. 5HTT primers (forward primer 5′-TCCGCATGAATGCTGTGTAAC-3′, reverse primer 5′-TTGGCTTAGAGGGGAGGAGTC-3, product of 102 bp) were used to quantitate total genomic DNA, and SRY primers were used to quantitate male genomic DNA, and the percentage of male genomic DNA was determined by dividing the quantity of male genomic DNA by the total genomic DNA. Each real-time PCR reaction included 1 × QuantiTect SYBR Green PCR Master Mix (Qiagen), 0.3 *μ*M of each primer, and 2 *μ*L of sample DNA or serially diluted standard male genomic DNA. The PCR program was 95°C for 15 minutes, 40 cycles of 94°C for 15 seconds, 57°C for 30 seconds, and 72°C for 30 seconds.

### 2.6. Statistical Analysis

Data are presented as mean values ± standard deviations. Survival analysis and intergroup comparisons were performed using ANOVA followed by the least-significant difference (LSD) or Bonferroni/Dunn Test, which compensated for unequal group size. *P* < .05 was considered statistically significant.

## 3. Results

### 3.1. The Morbidity of aGVHD and Animal Survival

The morbidity of aGVHD in different groups following liver transplantation is summarized in Table 1. In group 2, the (LewisXBN)F1 recipients that had received semiallogeneic liver transplantation without splenocyte transfusion all survived >100 days without any evidence of aGVHD, as did the animals in group 1, which received syngeneic liver transplantation. In groups 3 and 4, which received a splenocyte transfusion in addition to liver transplantation, the survival of rats diminished depending on the number of donor splenocytes transferred. In group 3, which received a transfusion of 2 × 10^8^ splenocytes, lethal aGVHD occurred in three recipients (50%), the whereas administration of 4 × 10^8^ splenocytes to group 4 led to an aGVHD morbidity rate of 100% (6/6). No sublethal aGVHD was observed in these groups.

### 3.2. The Clinical Course and Pathological Findings of aGVHD Cxmfzy

**Figure 1:**
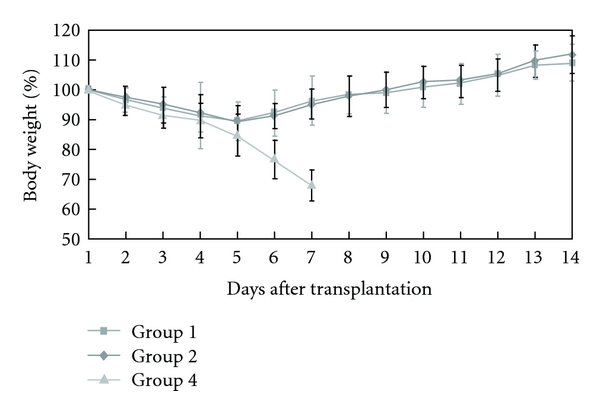
Postoperative body weight curves following liver transplantation. In group 1 and 2, all recipients lost body weight shortly after transplantation but regained weight and grew well thereafter. When the recipients developed aGVHD in group 4, they lost weight progressively.

**Figure 2:**
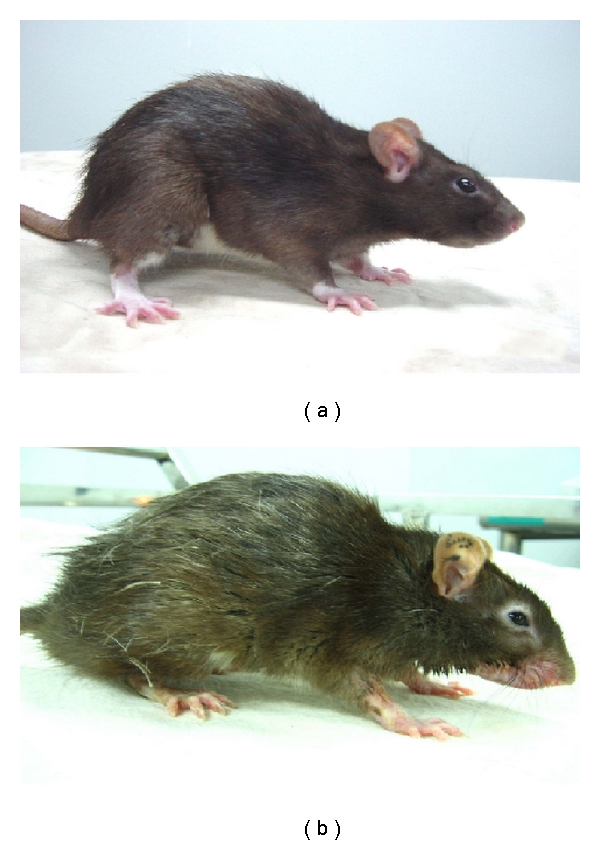
Clinical signs and pathological findings of aGVHD after liver transplantation. (a) In group 2, the appearance of the recipient returned to normal 2 weeks after transplantation. (b) On the 16th day after liver transplantation, (LewisXBN)F1 recipients in group 4 showed typical clinical signs of aGVHD, including diffuse alopecia (especially around the ear, eye, and food), severe athrepsy, hunched posture, and obvious cachectic.

After liver transplantation, all recipients had weight loss due to operative wound. In groups 1 and 2, the weight returned to the pretransplantation levels gradually. When the recipients developed aGVHD in group 4, they lost weight progressively (Figure 1). In group 2, the appearance of the recipients returned to normal gradually after the transplantation (Figure 2(a)). The clinical course of aGVHD was similar in groups 3 and 4. When a (LewisxBN)F1 recipient developed aGVHD, the first clinical signs of aGVHD appeared between days 7 and 10, manifested as severe dermatitis occurring predominantly on the ears, foot pads, and genitalia, then diffuse alopecia appeared. In the terminal stage of this disease, the rats suffered from diarrhea became increasingly cachectic, and exhibited a typical hunched posture (Figure 2(b)) that culminated in death from 19 to 33 days after transplantation. 

**Figure 3:**
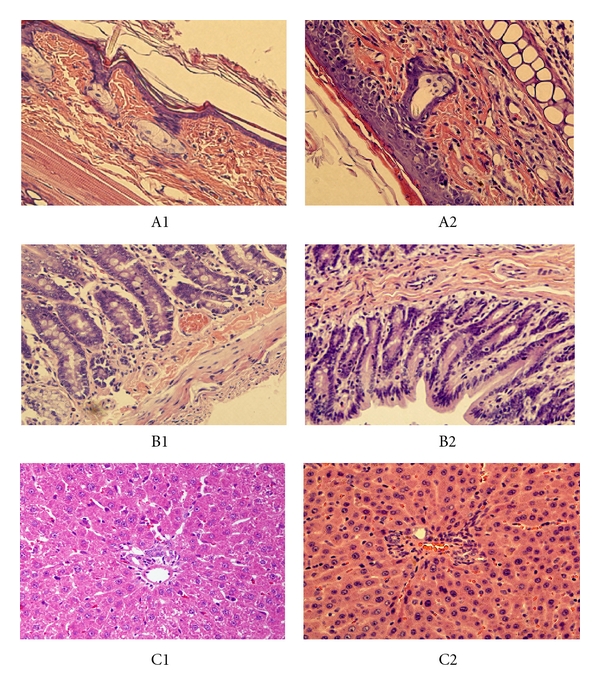
The pathological findings of aGVHD after liver transplantation. (A1) The histologic examination of skin was normal without obvious mononuclear infiltrating. (A2) Epidermis and dermis of the skin were infiltrated by mononuclear cells, and basilar degeneration and necrosis of keratinocytes were also observed. (B1) The histologic examination of intestine was normal without obvious mononuclear infiltrating. (B2) The intestine contained lymphocytic infiltrates. (C1) The histologic examination of liver was normal without obvious mononuclear infiltrating. (C2) No obvious mononuclear infiltrate in the liver. (H&E, original magnification ×400.)

Tissue samples were harvested at the time of death from the (LewisxBN)F1 recipients in groups 3 and 4 that developed aGVHD and also from separate groups sacrificed 16 days after transplantation. The histologic examination of skin, intestineas and liver was normal in group 2 (Figure 3-A1_,_ B1_,_ C1). In recipients that developed aGVHD in groups 3 and 4, the histologic examination of skin and intestine showed characteristic pathologic features. The epidermis and dermis in the skin were infiltrated by mononuclear cells. Basilar degeneration and necrosis of keratinocytes were also present (Figure 3-A2). The intestine contained villous atrophy and lymphocytic infiltrates (Figure 3-B2). Animals from the semiallogeneic group had normal liver grafts without obvious mononuclear infiltrate within portal tracts and sinusoids (Figure 3-C2).

### 3.3. Chimerism in Recipients after Syngeneic Liver Transplantation and Semiallogeneic Liver Transplantation

**Figure 4:**
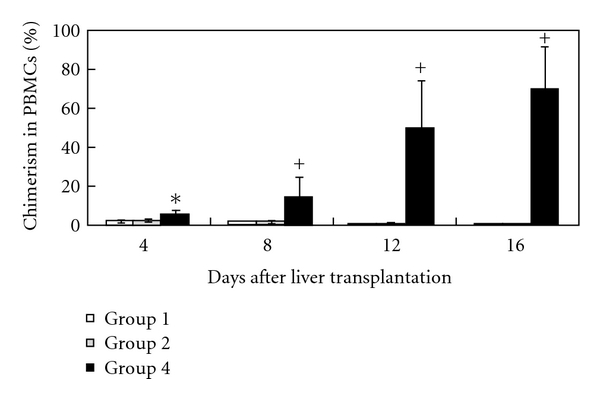
Changes of chimerism levels after liver transplantation. PBMC chimerism following sex-mismatched liver transplantation was identified by real-time PCR based on a rat Y-chromosome-specific primer, as described in Materials and Methods (Section  2). As described in Materials and Methods (Section  2), group 1 (white bars) received syngeneic liver transplantation, group 2 (gray bars) received semiallogeneic liver transplantation without splenocyte transfusion, and group 4 received semiallogeneic liver transplantation together with the transfusion of 4 × 10^8^ splenocytes. Each group consisted of 6 animals. *P < .05, ^+^ *P* < .01 (compared with groups 1 and 2).

The ratio of donor to recipient PBMCs after syngeneic liver transplantation (group 1) was 1.4%  ± 0.3%, 0.56%  ± 0.20%, 0.37%  ± 0.13%, 0.18%  ± 0.07%, and 0.02%  ± 0.02% on the 4th, 8th, 16th, 20th, and 50th day, respectively (shown as white bars in Figure 4).

The ratio of donor to recipient PBMCs after semiallogeneic liver transplantation (group 2) was 1.81%  ± 0.39% on the 4th day after transplantation, slightly higher than that of the group 1, but the difference was not statistically significant (*P* > .05). Then, the ratio of donor to recipient PBMCs decreased gradually to 1.03%  ± 0.42%, 0.69%  ± 0.26%, 0.39%  ± 0.29%, and 0.04%  ± 0.03%, on the 8th, 16th, 20th, and 50th day, respectively, which were not statistically different from the group 1 (*P* > .05) (shown as gray bars in Figure 4). No donor cells were detected 100 days after liver transplantation in both groups.

### 3.4. aGVHD after Liver Transplantation Is Associated with an Increased Level of Chimerism

In group 4, semiallogeneic liver transplantation together the with transfusion of 4 × 10^8^ splenocytes leads to the development of aGVHD in all recipients. By day 4 after transplantation, the ratio of donor to recipient PBMCs in these animals was 5.46%  ± 2.10%, significantly higher than those of groups 1 and 2 (*P* < .05). Unlike in groups 1 and 2, the level of chimerism in this group continued to elevate sharply afterwards, reaching 14.12%  ± 9.95%, 49.79%  ± 23.96%, and 69.68%  ± 21.97% on the 8th, 12th, and 16th day after transplantation, respectively. These values (shown as black bars in Figure 4) are significantly higher than those of groups 1 and 2 (*P* < .01).

### 3.5. The Influence of Splenocyte Transfusion on Chimerism after Liver Transplantation

**Figure 5:**
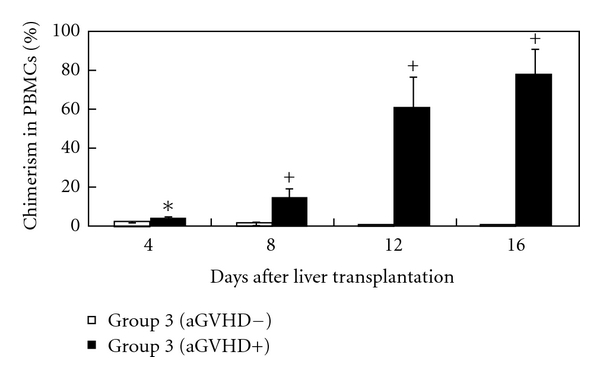
association of chimerism with aGVHD. PBMC chimerism following sex-mismatched liver transplantation was identified by real-time PCR based on a rat Y-chromosome-specific primer, as described in Materials and Methods (Section  2). As described in Materials and Methods (Section  2), group 3 received semiallogeneic liver transplantation together with the transfusion of 2 × 10^8^ splenocytes. Group 3 (aGVHD-), shown as white bars, consisted of 6 animals that were free from aGVHD, group 3 (aGVHD+) consisted of 6 animals with aGVHD following transplantation *P < .05, ^+^ *P* < .01.

The influence of splenocyte transfusion on chimerism was studied to confirm that high levels of chimerism observed in group 4 were correlated with the onset of aGVHD and not with the number of the splenocytes. Recipients in group 3 received a transfusion of 2 × 10^8^ splenocytes, resulting in 50% morbidity due to aGVHD (6/12 animals). Chimerism increased markedly in the recipients that developed aGVHD after transplantation (shown as black bars in Figure 5). Conversely, chimerism did not increase in the recipients without aGVHD, and between 4 and 100 days after the transplantation, the ratio of donor to recipient PBMCs did not differ significantly from that observed in groups 1 and 2 (shown as white bars in Figure 5).

## 4. Discussion

LTx-aGVHD results from the engraftment of T lymphocytes associated with the liver graft. However, monitoring the donor T-lymphocyte chimerism to aid in disease diagnosis is complicated by the fact that chimerism appears transiently in the majority of recipients after liver transplantation [19–21]. We showed that the presence of donor PBMCs increased transiently within the first several days after syngeneic liver transplantation and persisted for some time thereafter in the recipient's peripheral blood. Thereafter, chimerism declined rapidly and was usually absent beyond 100 days after transplantation. 

Donor-dominant one-way MHC matching (one-way matching between a MHC-homozygous donor and a haploidentical recipient) is a recognized risk factor for aGVHD following liver transplantation [30, 31]. This unidirectional transplant model allows for studies of the graft-versus-host reaction without the obscuring effects of a host-versus-graft reaction that leads to the rejection of donor lymphoid tissues which often occurs in a fully allogeneic transplant model [32]. 

However, even the liver contains large numbers of lymphoid cells in the parenchyma, the replacement of F1 liver with Lewis liver alone in the semiallogeneic group had virtually no influence on the recipient. They all survived indefinitely and grew in a normal pattern similar to that observed in the syngeneic liver transplantation, and there was no histological evidence of aGVHD. When chimerism was measured, no significant difference was observed in compaison with the syngeneic liver transplantation group. And beyond day 4, the ratio of donor to recipient PBMCs decreased gradually in both groups. These results indicate that the fate of donor cells in the recipient's peripheral blood was the same after semiallogeneic and syngeneic liver transplantation. It appears that mature donor cells may be eliminated very efficiently within days following a rapid migration into the recipient's circulation after transplantation.

In our previous study, the reproducible rat model of LTx-aGVHD has been established for the first time by performing LT from Lewis to (LewisXBN)F1 rat in combination with donor splenocyte transfusion [23]. And after the transfusion of 4 × 10^8^ donor splenocytes, simultaneous with liver transplantation, all recipients developed lethal aGVHD. The presence of chimerism in the PBMCs increased progressively after transplantation, and even at an early time (4 days after transplantation), a significant increase in chimerism was observed. Thus chimerism preceded the first clinical signs of aGVHD, which appeared between 7 and 10 days after liver transplantation. These results showed that elevated levels of chimerism are a strong predictor of aGVHD after liver transplantation, and the detection of chimerism may be of value in the diagnosis of aGVHD preceding the onset of clinical symptoms.

To confirm the value of chimerism as a predictor of aGVHD, it was necessary to rule out the influence of splenocyte transfusion on chimerism after liver transplantation in this LTx-aGVHD model. The incidence and morbidity of aGVHD depended on the number of splenocytes transferred: a dose of 4 × 10^8^ cells gave 100% morbidity, while 2 × 10^8^ cells gave only 50% death (groups 4 and 3, resp., in Table 1). Chimerism, however, correlated with the onset of aGVHD and not with the dose of splenocytes, in group 3, chimerism increased in the recipients that developed aGVHD after liver transplantation, whereas in the recipients without aGVHD, no significant differences were observed in comparison with groups 1 and 2 (Figures 1 and 2). These data suggest that the observed high levels of chimerism in groups 3 and 4 are caused by the aGVH reaction and not dependnt on splenocyte transfusion number.

Our results showed that the ratio of donor cells to recipient cells decreased gradually over time and that chimerism disappeared in group 1 and 2. This phenomenon may be explained by the induction of a weak immune response against the male-specific H-Y antigen after syngeneic sex-mismatched transplantation, because the male-specific H-Y antigen is known to be a minor histocompatibility antigen. The anti-H-Y immune response has been documented mainly as cytotoxicity mediated by cytotoxic T lymphocytes *in vitro* and by skin graft rejection *in vivo *[33–35]. In the nonimmunosuppressed sex-mismatched liver transplant recipients in our study, an immune response against the male-specific H-Y antigen may have caused the disappearance of donor cells from the blood stream without provoking a host-versus-graft reaction sufficient to induce rejection. Alternatively, the sex-mismatched graft might have induced mild, self-limiting rejection that recovered spontaneously.

Several studies have demonstrated that the fractionation of peripheral blood into subpopulations is useful for early detection of chimerism that may be undetectable in whole blood even when the percentage of aGVHD effector cells of donor origin is substantial [13, 16]. This fractionation can be achieved by fluorescent staining followed by flow cytometric sorting, and large numbers of lymphocyte subpopulations can be quickly and easily selected by sequential immunomagnetic beading. However, we did not analyze the chimerism in subpopulations of PBMCs in this study. Further experiments will be necessary to determine the subgroups of PBMCs responsible for aGVHD after liver transplantation.

In conclusion, we analyzed the change of chimerism in PBMCs following sex-mismatched liver transplantation by real-time PCR based on a rat Y-chromosome specific primer. And we demonstrated that the high-level chimerism correlated well with LTx-aGVHD, and the detection of chimerism soon after transplantation may be of value in the diagnosis of LTx-aGVHD prior to the onset of symptoms.

##  Funding Sources

National Natural Science Funds for Distinguished Young Scholar (no. 30925033), sponsored by Zhejiang Provincial Program for the Cultivation of High-level Innovative Health talents, and Natural Science Fund of Zhejiang Province (no. Y207420, no. Z2080283, and no. Y2090368).

##  Contributors

Fei Xue and Tingbo Liang designed this study, Fei Xue, Wei Chen, Xiaoguang Wang, and Liang liang performed this study, Xueli Bai and Linyan Wang: collected and analyzed the data, and Fei Xue wrote this paper.

## Figures and Tables

**Table 1:** Lethality of aGVHD, survival time of each individual animal, and mean survival time (MST) for each group following liver transplantation. Each transplantation group consisted of 6 animals.

Transplantation group	Number of donor splenocytes transferred	Lethality of aGVHD (fraction of total)	Survival time in days (number of animals)	MST in days
Group 1				
(L-L)	None	0	>100 (6)	>100
Group 2				
(L-F1)	None	0	>100 (6)	>100
Group 3				
(L-F1)	2 × 10^8^	50% (3/6)	20	62.5
			24	
			31	
			>100 (3)	
Group 4				
(L-F1)	4 × 10^8^	100% (6/6)	19	25.3
			21	
			24	
			26	
			29	
			33	

Abbreviations: MST: mean survival time; L-L: transplantation from Lewis rat to Lewis rat; L-F1: transplantation from Lewis rat to (LewisxBN)F1 rat.

## Abbreviations

aGVHD: Acute graft versus host disease
LTx-aGVHD: Acute graft versus host disease following liver transplantation
LT: Liver transplantation
MHC: Major histocompatibility complex
PBMCs: Peripheral blood mononuclear cells.
